# YAP1-mediated cytoplasmic-nuclear translocation of SREBP2 promotes colorectal cancer via regulation of cholesterol metabolism

**DOI:** 10.3892/ijo.2026.5918

**Published:** 2026-07-13

**Authors:** Luhui Zhong, Min Sun, Di Wu, Yulan Zeng, Min Zhou, Rui Gong, Feifei Gu, Kai Zhang, Xiaoyan Hu, Yue Hu

**Affiliations:** 1 Cancer Center, Union Hospital, Tongji Medical College, Huazhong University of Science and Technology, Wuhan, Hubei 430022, P.R. China; 2 Institute of Radiation Oncology, Union Hospital, Tongji Medical College, Huazhong University of Science and Technology, Wuhan, Hubei 430022, P.R. China; 3 Hubei Key Laboratory of Precision Radiation Oncology, Wuhan, Hubei 430022, P.R. China; 4 Research Center of Carcinogenesis and Targeted Therapy, Xiangya Hospital, Central South University, Changsha, Hunan 410008, P.R. China; 5 Department of Radiology, Union Hospital, Tongji Medical College, Huazhong University of Science and Technology, Wuhan, Hubei 430022, P.R. China; 6 Health Management Center, Union Hospital, Tongji Medical College, Huazhong University of Science and Technology, Wuhan, Hubei 430022, P.R. China; 7 Department of Radiation and Medical Oncology, Zhongnan Hospital of Wuhan University, Wuhan, Hubei 430071, P.R. China

**Keywords:** colorectal cancer, cholesterol metabolism, yes-associated protein 1, sterol regulatory element binding protein 2, cytoplasmic-nuclear translocation

## Abstract

Colorectal cancer (CRC) cells reprogram multiple metabolic pathways, including anaerobic glycolysis, lipogenesis and amino acid metabolism, to support their rapid cell division. However, the key regulators driving these metabolic alterations in CRC remain unknown. In the present study, immunohistochemistry staining of a tissue microarray demonstrated that yes-associated protein 1 (YAP1) was markedly upregulated in CRC tissues and strongly associated with worse patient survival. Knocking down YAP1 inhibited the proliferation and cholesterol accumulation of CRC cells both *in vitro* cell growth assays and *in vivo* xenograft model. Mechanistically, western blotting, co-immunoprecipitation and immunofluorescence assays showed that YAP1 not only transcriptionally upregulated sterol regulatory element binding protein 2 (SREBP2) expression but also physically interacted with it to facilitate its nuclear translocation. This coordinated regulation drove the expression of genes governing *de novo* cholesterol synthesis and exogenous cholesterol influx. Functional experiments revealed that the SREBP2-dependent cholesterol metabolic pathway was essential for YAP1-driven tumorigenesis and proliferation in CRC. These findings uncovered a YAP1-SREBP2 axis involved in CRC metabolic reprogramming and suggested that targeting this interaction may represent a promising metabolic intervention strategy for CRC management.

## Introduction

Colorectal cancer (CRC) is the second leading cause of cancer-related death globally, with ~20% of patients diagnosed in the first instance with metastatic CRC (1-3). Among the signaling cascades involved in CRC development and progression, the Hippo pathway has emerged as a critical regulator (4,5). As the primary downstream effector of the Hippo pathway, Yes-associated protein 1 (YAP1) orchestrates transcriptional programs governing cell proliferation, survival, and cell-fate decisions (6,7). YAP1 activity is predominantly regulated by phosphorylation of conserved S127 residue mediated by the large tumor suppressor kinase (LATS) 1/2. Upon phosphorylation, YAP1 is sequestered in the cytoplasm and targeted for proteasomal degradation, thereby suppressing its transcriptional activity (8,9). Beyond its classical functions, YAP1 has been increasingly recognized as a metabolic regulator, consistent with the fundamental importance of energy homeostasis in supporting cell growth and survival (10). In our previous study, it was demonstrated that YAP1 suppressed the expression of gluconeogenic genes in hepatocytes through peroxisome proliferator-activated receptor-γ coactivator-1, thereby redirecting metabolic resources from gluconeogenesis toward anabolic processes required for growth (11). In addition to glucose metabolism, YAP1 regulates glutaminolysis, a critical metabolic pathway that provides nitrogen and carbon for biosynthetic processes in cancer cells (12,13). Moreover, aberrantly elevated bile acids act as extrinsic signals that trigger YAP1 activation, leading to spontaneous liver tumorigenesis (14). The unsaturated fatty acids signal downstream of stearoyl-CoA desaturase 1, which positively promotes YAP1 expression in lung cancer stem cells (15). These studies suggest that YAP1 may act as the nexus between cancer cell growth and metabolism.

Lipids serve as essential structural components of cellular membranes, and their availability is critical for efficient cell proliferation. Cells that are rapidly dividing have a high demand for cholesterol to support their growth (10). Dysregulated cholesterol metabolism is now considered a hallmark of CRC, with supporting evidence from both clinical and preclinical studies. Hypercholesterolemia has been epidemiologically linked to elevated CRC risk, while long-term statin use is associated with reduced CRC incidence and mortality (16,17). In preclinical models, CRC cells transcriptionally reprogram cholesterol metabolism by upregulating genes involved in synthesis and uptake, driving proliferation, survival, and metastasis (18,19). A central regulator of this process is sterol regulatory element-binding protein 2 (SREBP2), a nuclear transcription factor that regulates the expression of genes critical for cholesterol homeostasis (20,21). In response to low sterol levels, the SREBP2 precursor is proteolytically cleaved, releasing its mature N-terminal domain, which enters the nucleus and modulates target gene expression (22). A previous study showed that YAP1 can functionally interact with SREBP1c/SREBP2 in a mouse model of diabetic liver, contributing to hepatic steatosis and hyperlipidemia (23). Additionally, YAP1 has been implicated in a lipogenic program in non-transformed MCF10A cells, where it acts through the SGK1-mTORC1-SREBP1 axis to promote proliferation and tissue expansion (24). Despite these insights, it remains uncertain whether YAP1 directly regulates cholesterol metabolism via SREBP2 in CRC, and if so, by what mechanism.

In the present study, SREBP2 was identified as a critical interacting partner and downstream effector of YAP1 in CRC cells. Abnormal elevation of YAP1 enhanced cholesterol uptake and de novo synthesis in CRC cells by interacting with SREBP2 and promoting its nuclear translocation. It was also demonstrated that inhibition of cholesterol metabolism suppressed YAP1-dependent tumorigenesis and proliferation. Taken together, these findings revealed that YAP1 regulated SREBP2-mediated cholesterol metabolism, thereby promoting CRC tumor growth.

## Materials and methods

### Cell culture

Human colorectal tumor cell lines HCT116 (ATCC^®^ CCL-247™) and SW480 (ATCC^®^ CCL-228™), as well as 293T (ATCC^®^ CRL-3216™) cells, were sourced from the ATCC. Cell line identities were confirmed by STR profiling within the past 3 years and all lines were routinely verified to be free of mycoplasma contamination using PCR. HCT116 and SW480 cells were maintained in RPMI-1640 medium, while 293T cells were maintained in DMEM. Both media were supplemented with 10% FBS (Gibco; Thermo Fisher Scientific, Inc.), and all cultures were maintained in a humidified incubator at 37°C supplied with air with 5% CO_2_.

### Plasmid and lentivirus infection

The short hairpin (sh) RNA constructs (shYAP1#1, shYAP1#2, shSREBP2, and shControl) and overexpression plasmids (oeYAP1, oeSREBP2, and oeYAP1 S127A) were generated and provided by Shanghai GeneChem Co., Ltd. For lentivirus production, second-generation packaging system was used. 293T cells were co-transfected with 6 *μ*g of target shRNA or overexpression plasmids and two packaging plasmids (4.5 *μ*g of pSPAX2 and 1.5 *μ*g of pMD2.G), using 35 *μ*l of PEI 25K (Polysciences, Inc.) as the transfection reagent. The cells were cultured continuously for 3 days at 37°C. To obtain the lentivirus, the supernatant was collected and subsequently filtered using a 0.45 *μ*m sterile filter. The resulting lentiviral particles were mixed with transfection enhancer reagent and then added to the target CRC cells (HCT116 and SW480) at a MOI of 10. Cells were transduced for 24 h, followed by a second round of transduction for another 24 h. Stable clones were selected using puromycin (MilliporeSigma) for 1 week, after which cells were collected for downstream experiments.

The shRNA sequences were: shControl: 5'-TTCTCCGAACGTGTCACGT-3'; shYAP1#1: 5'-TCAGAGTGCTCCAGTGAAA-3'; shYAP1#2: 5'-GGTCAGAGATACTTCTTAA-3'; and shSREBP2: 5'-GCCCTCTATTGGATGATGCAA-3'.

### Reverse-transcription quantitative (RT-q) PCR

Total RNA was isolated from cells at 80-90% confluence using the Omega Total RNA extraction kit (Yuanmu Bio-Technology) and reverse transcribed using HiScript III All-in-one RT SuperMix (Vazyme Biotech Co., Ltd.) according to the manufacturer's protocol. Subsequently, PCR amplification was performed using Taq Pro Universal SYBR qPCR Master Mix (Vazyme Biotech Co., Ltd.) according to the manufacturer's protocol. The cycling conditions were: Initial denaturation at 95°C for 3 min, followed by 40 cycles of denaturation at 95°C for 10 sec and annealing and extension at 60°C for 30 sec. Gene expression was quantified using the 2^−ΔΔCq^ method (25). The primer sequences are listed in Table I.

### Cell growth assays

Cell viability was assessed using the Cell Counting Kit-8 (CCK-8) solution (Biosharp Life Sciences) according to the manufacturer's protocol. Absorbance at 450 nm was measured at the indicated time points using a microplate reader (PerkinElmer, Inc.) to plot growth curves. Colony formation was assessed using crystal violet staining (Biosharp Life Sciences) at room temperature for 1 h following 2 weeks of culture of cells initially plated at 500 per well in six-well plates. Colonies containing >50 cells were counted. An EdU incorporation for DNA synthesis detection was performed using the BeyoClick EdU Kit (Beyotime Biotechnology) according to the manufacturer's protocol. A total of 5×10^3^ cells/well were plated in 96-well plates, stained, and imaged using an Olympus 5IX71 fluorescence microscope (Olympus Corporation).

### Tissue cell cholesterol level measurement

Cholesterol was extracted from cell pellets or minced tissues derived from mouse xenografts using the Tissue Cell Total Cholesterol Assay Kit (Beijing Pulilai Gene Technology Co., Ltd.) according to the manufacturer's protocol. The cleared supernatants were heated at 70°C, centrifuged at 2,000 × g for 5 min, and incubated with the working solution for 20 min. Subsequently, the absorbance was measured at 550 nm. Cholesterol levels were standardized using total protein as a reference.

### Triglyceride quantification

Triglyceride levels were measured using the triglyceride assay kit (Beijing Solarbio Science & Technology Co., Ltd.) according to the manufacturer's protocol. The cleared supernatants were incubated with the working solutions at 65°C for 15 min, and the absorbance was measured at 420 nm. Triglyceride levels were normalized to total protein concentration.

### Nile Red lipid droplet staining

To visualize lipid droplets, cells at 80-90% confluence grown in 24-well plates were fixed with a lipid fixative for 15 min at room temperature, washed with PBS, and stained using a Lipid Fluorescence Staining Kit (Beijing Solarbio Science & Technology Co., Ltd.) according to the manufacturer's protocol. Nuclei were counterstained with DAPI at room temperature for 5 min. Under light-proof conditions, images were captured within 30 min using a fluorescence microscope. Cellular lipids appeared as red or bright orange fluorescence, while the cell nucleus was stained bright blue with DAPI.

### LC-MS/MS analysis of metabolomics

CRC cells were transfected with shRNA lentiviral vectors targeting YAP1. The cultured cell medium was obtained by centrifugation and used for UPLC-MS/MS analysis. The UPLC-MS/MS system consisted of an ExionLC AD ultra-performance liquid chromatography system (SCIEX) coupled to a QTRAP tandem mass spectrometer (SCIEX). Mass spectrometry was performed using an electrospray ionization source in both positive and negative ion modes. The ion source temperature was set to 500°C; the nebulizer gas (GS1) was set to 45-55 psi, the auxiliary gas (GS2) to 55-60 psi, and the curtain gas to 25-35 psi. Multiple reaction monitoring was used to monitor precursor-to-product ion transitions, with declustering potential and collision energy optimized for each transition. Qualitative analysis of metabolites was conducted based on retention time, and quantitative analysis was performed using multiple reaction monitoring. The metabolite data were normalized using unit-variance scaling (Z-score). To visualize the hierarchical clustering of samples and metabolites, a heatmap was generated using the Complex Heatmap package (version 2.8.0; https://bioconductor.org/packages/ComplexHeatmap) in R software (version 4.1.2). In the two-group comparison, differentially abundant metabolites were selected based on Variable Importance in Projection (VIP≥1) and absolute Log_2_ Fold Change (|Log_2_FC|≥1.0). Subsequently, these metabolites were analyzed using KEGG pathway annotation and mapping (kegg.jp/kegg/pathway.html).

### RNA sequencing (RNA-seq)

Total RNA was extracted from HCT116 cells transfected with shYAP1 or control shRNA, using TRIzol reagent (Invitrogen; Thermo Fisher Scientific, Inc.; cat. no. 15596026). RNA quality and integrity were assessed using the RNA 6000 Nano LabChip Kit (Agilent Technologies; cat. no. 5067-1511) on an Agilent 2100 Bioanalyzer (Agilent Technologies; cat. no. G2939BA) according to the manufacturer's protocol. RNA-seq library was prepared using the VAHTS Universal V8 RNA-seq Library Prep Kit for Illumina (Vazyme Biotech Co., Ltd.; cat. no. NR605-01). Sequencing was performed by Wuhan Metware Biotechnology Co., Ltd. using an Illumina NovaSeq 6000 platform with a NovaSeq 6000 SP Reagent Kit (Illumina Inc.; cat. no. 20028401). The final library concentration was >2 nM as measured by a Qubit 2.0 fluorometer (Thermo Fisher Scientific, Inc.) and was diluted to approximately 1.8 pM for loading onto the flow cell according to the Illumina protocol. Paired-end sequencing (150 bp) was performed. Raw sequencing reads were processed and aligned to the human reference genome (GRCh38) using standard pipelines. Gene expression levels were quantified, and differential expression analysis was performed using DESeq2 (version 1.32.0; https://bioconductor.org/packages/DESeq2). Genes with |log_2_FC|≥1 and FDR<0.05 were considered differentially expressed.

### Gene set enrichment analysis (GSEA)

GSEA was performed using the fgsea package (version 1.24.0; https://bioconductor.org/packages/fgsea) in R software (version 4.1.2) to identify biological pathways associated with YAP1 knockdown. Genes were ranked by the Log_2_FC. The analysis was conducted using the Molecular Signatures Database (MSigDB) hallmark gene set (version 7.5; https://www.gsea-msigdb.org/gsea/msigdb/) with 1,000 gene set permutations. Gene sets with a normalized enrichment score (|NES|>1.5) and FDR<0.25 were considered significantly enriched.

### Western blotting

Cells were lysed using RIPA lysis buffer containing protease inhibitor and phosphatase inhibitor cocktails (Beyotime Biotechnology). Protein concentration was determined using the BCA protein assay kit (Beyotime Biotechnology) according to the manufacturer's protocol. The 8-12% resolving gel for SDS-PAGE was selected based on the protein's molecular weight, and 20-40 *μ*g of total protein extract were loaded per well. The separated protein samples were transferred to PVDF membranes (MilliporeSigma). After blocking with 5% skimmed milk for 1 h at room temperature, membranes were incubated overnight at 4°C with primary antibodies, followed by HRP-conjugated secondary antibodies for 1 h at room temperature. Signals were visualized using enhanced chemiluminescence reagent (Dalian Meilun Biology Technology Co., Ltd.). Densitometry analysis was performed using ImageJ version 1.54f (National Institutes of Health), and signals were normalized to the corresponding loading controls. Relative protein levels were calculated as fold change compared to control samples. The antibodies used for western lot are listed in Table II.

### Co-immunoprecipitation

For endogenous protein-binding experiments, cells were lysed on ice in RIPA lysis buffer containing protease inhibitor and phosphatase inhibitor cocktails (Beyotime Biotechnology), then centrifuged at 12,000 × g for 20 min at 4°C to pellet debris. Supernatants were incubated overnight at 4°C with 30 *μ*l prepared Protein A/G Plus-Agarose beads (MilliporeSigma) and a diluent containing IgG (Santa Cruz Biotechnology, Inc.; cat. no. sc-515946; 1:10,000) or YAP1 antibodies (ProteinTech Group, Inc.; cat. no. 13584-1-AP; 1 *μ*g for 3 mg of total protein lysate). The following day, the samples were washed with NETN buffer, centrifuged at 10,000 × g for 1 min at 4°C, and the isolated immunoprecipitated proteins were denatured at 100°C and analyzed by western blotting. For exogenous protein binding experiments, YAP1-Flag and SREBP2-Myc expression plasmids constructed on the pcDNA3.1 backbone were purchased from Wuhan GeneCreate Biological Engineering Co., Ltd and co-transfected into 293T cells. Anti-Flag (ABclonal Biotechnology Co., Ltd.; cat. no. AE005; 5 *μ*g/ml cell lysate) and anti-Myc (ABclonal Biotechnology Co., Ltd.; cat. no. AE070; 5 *μ*g/ml cell lysate) immunomagnetic beads were pretreated and used. An appropriate amount of the centrifuged protein supernatant was reserved as the input sample, while the residual lysate was mixed with immunomagnetic beads and agitated at 4°C overnight. The mixture was washed using a magnetic frame and denatured at 100°C to obtain IP samples, which were then analyzed by western blotting.

### Cytoplasmic and nuclear protein extraction

Nuclear and cytoplasmic fractions were isolated from cells using the Nuclear and Cytoplasmic Protein Extraction Kit (Beijing Solarbio Science & Technology Co., Ltd.), according to the manufacturer's protocol. The resulting protein samples were then analyzed by western blotting, with Histone H3 as the nuclear reference protein.

### Immunohistochemistry (IHC) staining of the tissue microarray

The human CRC tissue microarray (HColA180Su20) used in the present study was obtained from Shanghai Outdo Biotech Co., Ltd. The tissue microarray consisted of 180 tissue points, including 96 cases of CRC tissues and 84 matched non-neoplastic normal tissues, all collected from surgical specimens. Due to the technical limitations inherent to tissue microarrays (for example, insufficient evaluable tissue), clinicopathological data were not available for all patients. The number of patients with available data for each parameter (age, sex, histological type, histological grade, disease stage, T stage, N stage and M stage) is specified in Table III. Staging of the tumor was conducted in accordance with the AJCC 8th Edition recommendations (26). For immunohistochemical staining, endogenous peroxidase activity was blocked with 3% H_2_O_2_ for 10 min at room temperature. Tissue microarray was permeabilized with 0.1% Triton X-100 in PBS for 10 min, blocked with 10% normal goat serum (Beyotime Biotechnology) for 10 min at room temperature, and washed with PBS. Tissue microarray was incubated overnight at 4°C with anti-YAP1 antibody (ProteinTech Group, Inc.; cat. no. 66900-1-Ig; 1:1,500), washed with PBS, and then incubated with HRP-conjugated goat anti-mouse IgG (Wuhan Servicebio Technology Co., Ltd.; cat. no. GB23301; 1:1,000) for 1 h at room temperature. The signal was visualized using DAB substrate solution (Beyotime Biotechnology). Tissue microarray was counterstained with hematoxylin for 2 min at room temperature, dehydrated, and mounted. Images were captured using an Olympus BX51 light microscope (Olympus Corporation). IHC intensity was graded as follows: Negative, no staining of cells=0; Low, staining=1; Medium, staining=2; High, staining=3. The percentage of stained area in tissues was divided based on the number of positive-staining cells as follows: 0-25%=1, 26-50%=2, 51-75%=3, >75%=4. The YAP1 staining index in CRC tissues was calculated by multiplying the staining intensity score by the percentage of stained area score. A staining index of ≥6 indicated high YAP1 expression, while scores <6 denoted low expression.

### Immunofluorescence (IF) staining

For cultured cells, cells (2×10^5^ cells/well) grown on the glass coverslips in 24-well plates were fixed with 4% paraformaldehyde for 20 min at room temperature, permeabilized with 0.2% Triton X-100 and blocked with 5% BSA solution for 1 h. After washing with PBS, the primary antibodies were diluted in 1% BSA solution and incubated overnight at 4°C with the processed cells. The primary antibodies used were anti-YAP1 (ProteinTech Group, Inc.; cat. no. 13584-1-AP; 1:200), anti-phospho-YAP1 S127 (Assay Genie; cat. no. CABP0489; 1:100), and anti-SREBP2 (Santa Cruz Biotechnology, Inc.; cat. no. sc-271615, 1:300). The fluorescent secondary antibodies used were: FITC-conjugated antibody (Wuhan Servicebio Technology Co., Ltd.; cat. no. GB22401/GB22403; 1:50) or CY3-conjugated antibody (Wuhan Servicebio Technology Co., Ltd.; cat. no. GB21301/GB21303, 1:100). After incubation with the primary antibodies and washing with PBS, the secondary antibodies were added to cells and cultured in the dark for 1 h at room temperature. Subsequently, nuclei were counterstained with DAPI for 10 min at room temperature. The glass coverslips were mounted with anti-fade mounting (Beyotime Biotechnology). For human tissue sections, samples from three patients diagnosed with CRC (two males and one female; age, 49, 55, and 69 years) were obtained from Union Hospital, Tongji Medical College, Huazhong University of Science and Technology (Wuhan, China). Formalin-fixed paraffin-embedded samples were deparaffinized, rehydrated, and subjected to heat-induced antigen retrieval. Following blocking with 10% normal donkey serum (Wuhan Antgene Biotechnology Co., Ltd.), the sections were incubated with primary antibodies against YAP1 (ProteinTech Group, Inc.; cat. no. 66900-1-Ig; 1:1,000) or SREBF2 (ProteinTech Group, Inc.; cat. no. 28212-1-AP; 1:100). After washing, sections were incubated with Alexa Fluor 488-conjugated donkey anti-mouse (Thermo Fisher Scientific, Inc.; cat. no. A21206; 1:400) or Alexa Fluor 647-conjugated donkey anti-rabbit secondary antibodies (Abcam; cat. no. ab150075; 1:400) at room temperature. DAPI was used to counterstain the nuclei for 10 min at room temperature. All fluorescence images were acquired using a confocal laser scanning microscope (Leica TCS SP8; Leica GmbH) at ×200 and ×400 magnification.

### Mouse CRC model

All animal experiments were approved by the Institutional Animal Care and Use Committee of Huazhong University of Science and Technology (approval no. 4588). A total of six-week-old male BALB/c nude mice (18-22 g) were purchased from the Wuhan Beisai Model Biotechnology Co., Ltd. and housed under SPF conditions at 21-24°C with 50-60% humidity, a 12 h light-dark cycle and free access to food and water. Stably transfected HCT116 cells (5×10^6^ cells in 100 *μ*l PBS) were subcutaneously injected into the right hind limb of each mouse under anesthesia induced by intraperitoneal injection of pentobarbital sodium (50 mg/kg body weight). The long and short diameters of the subcutaneously transplanted tumors were measured every 3 days starting from 2 weeks post-injection. The tumor volume was calculated using the following formula: Volume=(a × b^2^)/2 where a and b represent the long and short diameter, respectively. In strict accordance with the animal ethics guidelines, the tumor weight did not exceed 10% of the body weight, the mean tumor diameter did not exceed 20 mm and the tumor volume did not exceed 1,000 mm^3^. The maximum tumor volume recorded was 923.5 mm^3^, and the maximum tumor diameter was 17 mm. At the experimental endpoint, mice were sacrificed by cervical dislocation under isoflurane anesthesia (5% for induction and 2% for maintenance) and mortality was confirmed by respiratory arrest and lack of heartbeat. All procedures were performed in accordance with institutional guidelines to minimize suffering.

### Statistical analysis

Microsoft Excel (Microsoft Corporation) and ImageJ version 1.54f (National Institutes of Health) were used for data processing and graphs were generated using GraphPad version Prism 9.0 (Dotmatics). Statistical comparisons between groups were conducted using a two-tailed unpaired t-test, one-way ANOVA followed by Tukey's post-hoc test, Pearson χ^2^ test, or a Fisher's exact test. P<0.05 was considered to indicate a statistically significant difference.

## Results

### YAP1 is markedly upregulated in colorectal carcinoma

**Figure 1:**
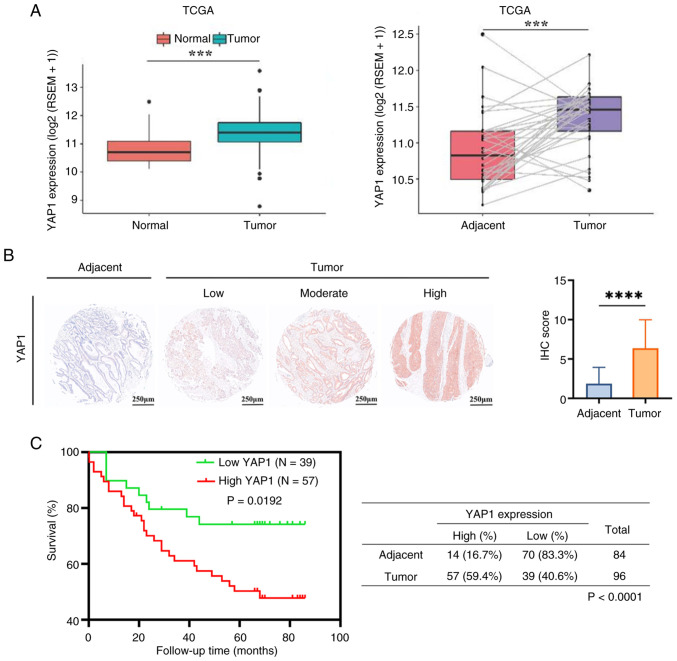
YAP1 expression in CRC and adjacent normal tissues. (A) Analysis of TCGA RNA-seq data showed higher YAP1 mRNA levels in CRC (n=383) compared to non-tumor tissues (n=51) and matched adjacent normal samples (n=32 pairs). (B) Representative IHC staining of YAP1 in a CRC tissue microarray, with corresponding quantification. Scale bar, 250 *μ*m. (C) Kaplan-Meier survival curve for overall survival based on YAP1 expression in 96 CRC cases. ^***^P<0.001, ^****^P<0.0001. YAP1, yes-associated protein 1; CRC, colorectal cancer; TCGA, The Cancer Genome Atlas; RNA-seq, RNA sequencing; IHC, immunohistochemistry.

To determine YAP1 levels in CRC, publicly available RNA-seq data from TCGA were analyzed. Compared with normal colorectal epithelium and adjacent non-cancerous tissues, CRC specimens showed substantially increased YAP1 mRNA expression (Fig. 1A). YAP1 protein expression was assessed using IHC staining on a tissue microarray containing paired CRC and adjacent normal samples. YAP1 expression was markedly stronger in CRC tissues than in their matched non-tumor counterparts (Figs. 1B and S1). Elevated YAP1 expression was also markedly associated with histological type (Table III).

Next, whether YAP1 protein levels were associated with patient survival in the same cohort of 96 CRC cases was examined. Kaplan-Meier analysis demonstrated that patients with increased YAP1 expression had markedly shorter overall survival (Fig. 1C). To assess whether this prognostic effect was independent of other clinical variables, multivariate Cox regression analysis was performed. After controlling for disease stage, age, sex, histological grade and histological type, high YAP1 expression retained its independent prognostic significance [Hazard ratio (HR)=2.322, 95% Confidence Interval (CI): 1.122-4.807; P=0.023; Fig. S2 and Table IV]. Stratified analysis by disease stage further confirmed the prognostic value of YAP1. The association between high YAP1 expression and poor survival was significant in patients with advanced-stage disease (Fig. S3B), but not in those with early-stage CRC (Fig. S3A). These findings indicate that the prognostic impact of YAP1 is more pronounced in advanced-stage CRC.

Collectively, these data showed that YAP1 upregulation in CRC was associated with poor clinical outcomes, particularly in patients with advanced disease.

### Knockdown of YAP1 inhibits colorectal tumor cell proliferation

**Figure 2:**
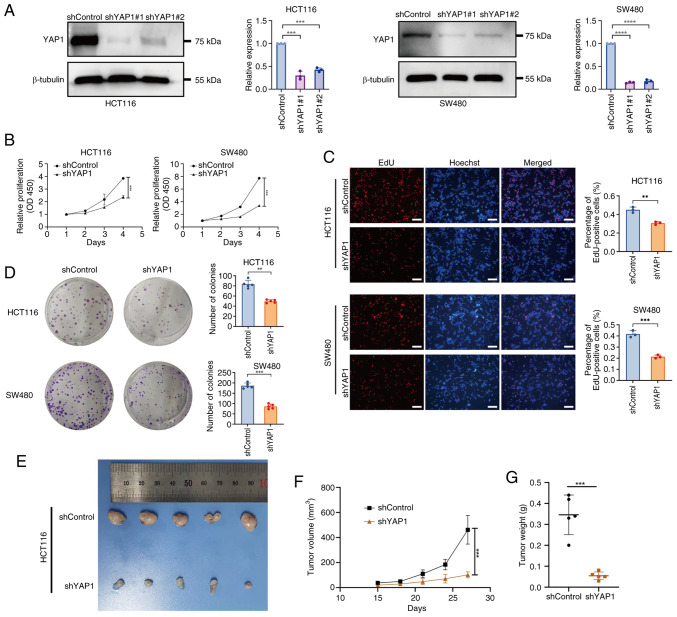
YAP1 promotes tumorigenic potential of CRC cells *in vitro* and *in vivo*. (A) Western blotting confirmation of YAP1 knockdown in HCT116 and SW480 cells transduced with lentiviral shRNAs (shYAP1#1 and shYAP1#2). β-Tubulin was used as the loading control. (B) CCK-8 assays showed reduced viability of YAP1-knockdown HCT116 and SW480 cells. (C) EdU incorporation assays demonstrated decreased proliferation following YAP1 knockdown. Scale bar, 100 *μ*m. (D) Colony formation assays revealed reduced colony formation capacity of YAP1-knockdown CRC cells. Representative images and colony counts are presented. *In vivo* tumor growth following subcutaneous injection of control or YAP1-knockdown HCT116 cells into nude mice (n=5 per group). (E) Tumor morphological images. (F) Tumor volumes were measured at the indicated time points. (G) Tumor weights at day 27 post-injection. All quantitative results are represented mean ± standard deviation of at least three separate experiments. ^**^P<0.01, ^***^P<0.001, ^****^P<0.0001. YAP1, yes-associated protein 1; CRC, colorectal cancer; shRNA, short hairpin RNA.

To investigate the functional significance of YAP1 in CRC progression, HCT116 and SW480 cells were transfected with shRNA lentivirus vectors targeting YAP1. The effectiveness of YAP1 knockdown was verified by western blotting (Fig. 2A). Although both shRNA sequences effectively reduced YAP1 levels, shYAP1#1(hereafter referred to as shYAP1) exhibited improved knockdown efficiency and was selected for subsequent experiments. YAP1 knockdown resulted in a notable decrease in the viability of both HCT116 and SW480 cells based on the CCK-8 assay (Fig 2B). The inhibitory effect of YAP1 knockdown on cell proliferation was further corroborated by a significant decrease in the number of EdU-positive cells following YAP1 knockdown (Fig. 2C). Furthermore, the colony-forming capacity of HCT116 and SW480 cells was markedly lower in the YAP1 knockdown cells (Fig. 2D). These findings indicated that YAP1 was involved in CRC cell proliferation and colony formation survival.

Extending these findings to an *in vivo* setting, control and YAP1-silenced HCT116 cells were subcutaneously injected into the right hind limbs of nude mice. After 2 weeks, tumor growth was monitored, and tumor sizes were quantified every 3 days. Consistent with the *in vitro* results, mice injected with YAP1-knockdown cells exhibited a significant reduction in tumor growth (Fig. 2E-G). Together, these results showed that reduced YAP1 expression could potentially inhibit CRC tumor growth *in vivo* and *in vitro*.

### YAP1 potentiates CRC cholesterol biosynthesis

**Figure 3:**
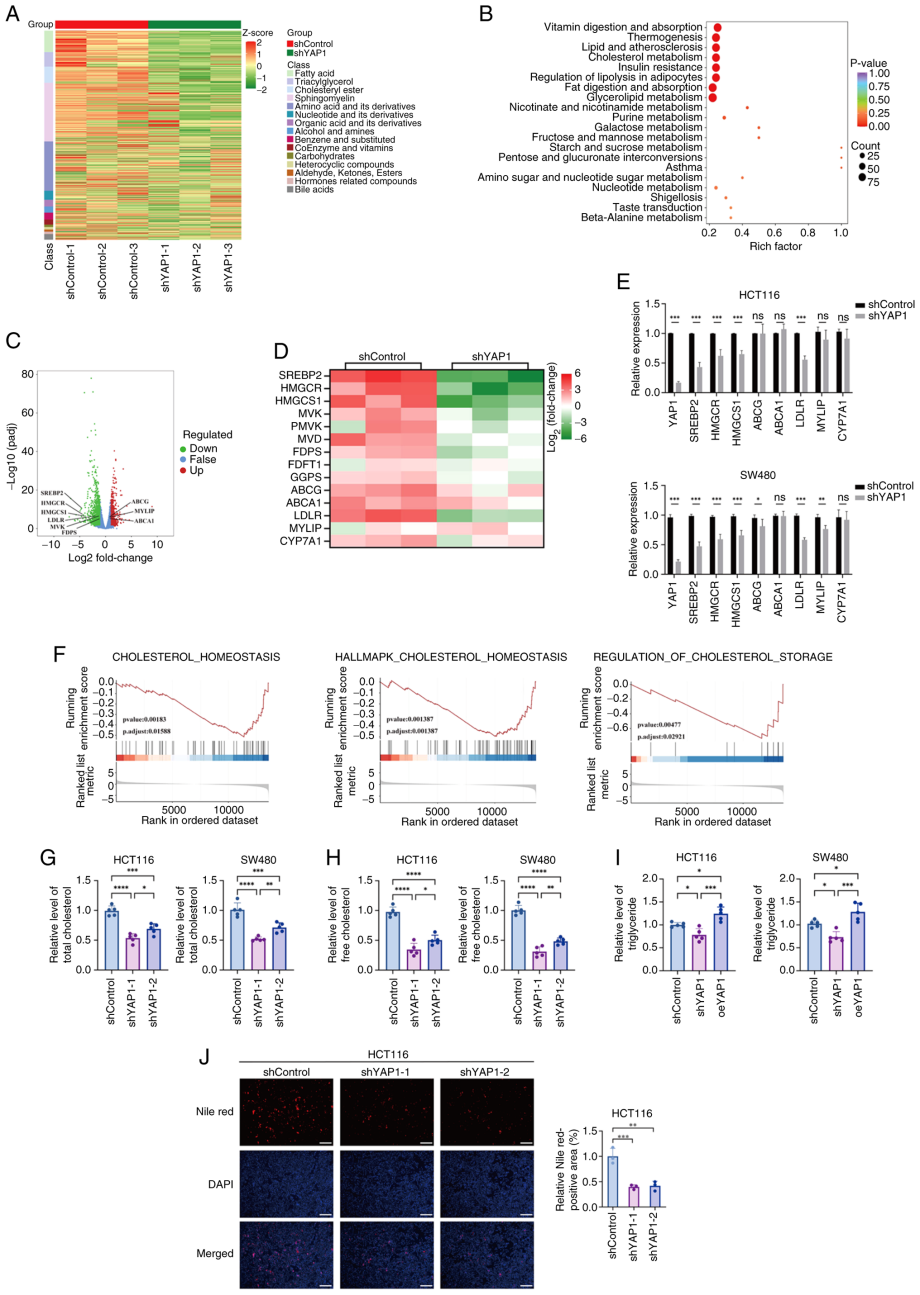
YAP1 regulates cholesterol metabolism in CRC cells. (A) Hierarchical clustering of differentially abundant metabolites in YAP1-knockdown vs. control HCT116 cells. (B) KEGG pathway analysis was used to identify the top downregulated metabolic pathways following YAP1 knockdown (n=3 per group). (C) Volcano plot showing the differentially expressed genes identified by RNA-seq in shYAP1 vs. shControl HCT116 cells. Genes involved in cholesterol homeostasis are highlighted. (D) Heatmap showing the relative expression levels of cholesterol metabolism genes in control and YAP1-knockdown HCT116 cells (n=3 per group). (E) Reverse transcription-quantitative PCR validation of cholesterol metabolism gene expression in YAP1-knockdown HCT116 and SW480 cells. (F) GSEA showing downregulation of cholesterol homeostasis and cholesterol storage signaling in shYAP1 HCT116 cells. (G) Total cholesterol and (H) free cholesterol levels in control and YAP1-knockdown HCT116 and SW480 cells. (I) Triglyceride contents in control, YAP1-knockdown and YAP1-overexpressing HCT116 and SW480 cells. (J) Nile Red staining showing lipid droplet accumulation following YAP1-knockdown, with quantification of the relative Nile Red-positive area. Scale bar, 100 *μ*m. ^*^P<0.05, ^**^P<0.01, ^***^P<0.001, ^****^P<0.0001. ns, not significant; YAP1, yes-associated protein 1; CRC, colorectal cancer; KEGG, Kyoto Encyclopedia of Genes and Genomes; shYAP1, short hairpin RNA targeting YAP1; oeYAP1, YAP1 overexpression; shControl, short hairpin RNA control; GSEA, Gene Set Enrichment Analysis.

Uncontrolled proliferation in cancer is accompanied by profound alterations in cellular metabolism to ensure an adequate supply of building blocks. To investigate how YAP1 promoted CRC cell proliferation at the metabolic level, the metabolic profiles of shControl and shYAP1 HCT116 cells using quantitative LC-MS metabolomics were compared. Untargeted metabolomics analysis was performed to determine global metabolite changes in YAP1-silencing HCT116 cells. In HCT116 cells with YAP1 knockdown, the concentrations of steroid compounds, including cholesterol esters, vitamins, bile acids, and hormones-related substances, were decreased compared to the shControl cells (Fig. 3A). Furthermore, upon YAP1 knockdown, the KEGG pathway enrichment analysis further showed a pronounced influence on pathways associated with vitamin digestion and absorption, along with lipid and atherosclerosis, and cholesterol homeostasis (Fig. 3B), which suggested a reduction in cholesterol levels upon YAP1 suppression.

RNA-seq was next performed on YAP1-silenced HCT116 cells to assess the transcriptional changes underlying the observed metabolic alterations. Differential expression analysis of RNA-seq data identified 676 upregulated and 1,261 downregulated genes in YAP1-knockdown cells compared to the control (Fig. 3C). Among the downregulated genes, there was a marked reduction in transcripts encoding key enzymes of the cholesterol biosynthetic pathway, including *SREBP2*, *HMGCR*, *HMGCS*, *MVK*, *PMVK*, *MVD*, *FDPS*, *FDFT1* and *GGPS* (Fig. 3D and E). Additionally, the gene *LDLR*, which is involved in cholesterol uptake, exhibited moderate downregulation (Fig. 3D and E). In contrast, the bile acid synthesis gene *CYP7A1*, cholesterol uptake suppression gene *MYLIP*, and cholesterol efflux genes including *ABCG* and *ABCA1*, did not exhibit notable changes in expression (Fig. 3D and E). Gene set enrichment analysis (GSEA) further confirmed that pathways related to cholesterol homeostasis and cholesterol storage were suppressed in YAP1-knockdown cells (Fig. 3F). These results indicated that YAP1 maintained cholesterol homeostasis of CRC cells predominantly by modulating *de novo* cholesterol synthesis and uptake pathways rather than by influencing the cholesterol efflux pathway.

To test directly whether YAP1 regulated cholesterol metabolism, lipid accumulation and cholesterol content in YAP1-knockdown HCT116 and SW480 cells were assessed. The assays confirmed that YAP1 knockdown led to a significant reduction in both total and free cholesterol levels in CRC cells (Fig. 3G and H). In addition, YAP1 knockdown also decreased cellular triglyceride levels, while YAP1 overexpression increased triglyceride content (Fig. 3I), indicating a broader regulatory role of YAP1 in neutral lipid metabolism. Nile Red staining further showed that YAP1 knockdown reduced lipid droplet accumulation in CRC cells (Fig. 3J), consistent with the reduced cholesteryl ester storage. Collectively, these findings showed that YAP1 regulated cholesterol metabolism in CRC cells.

### YAP1 reciprocally interacts with SREBP2

**Figure 4:**
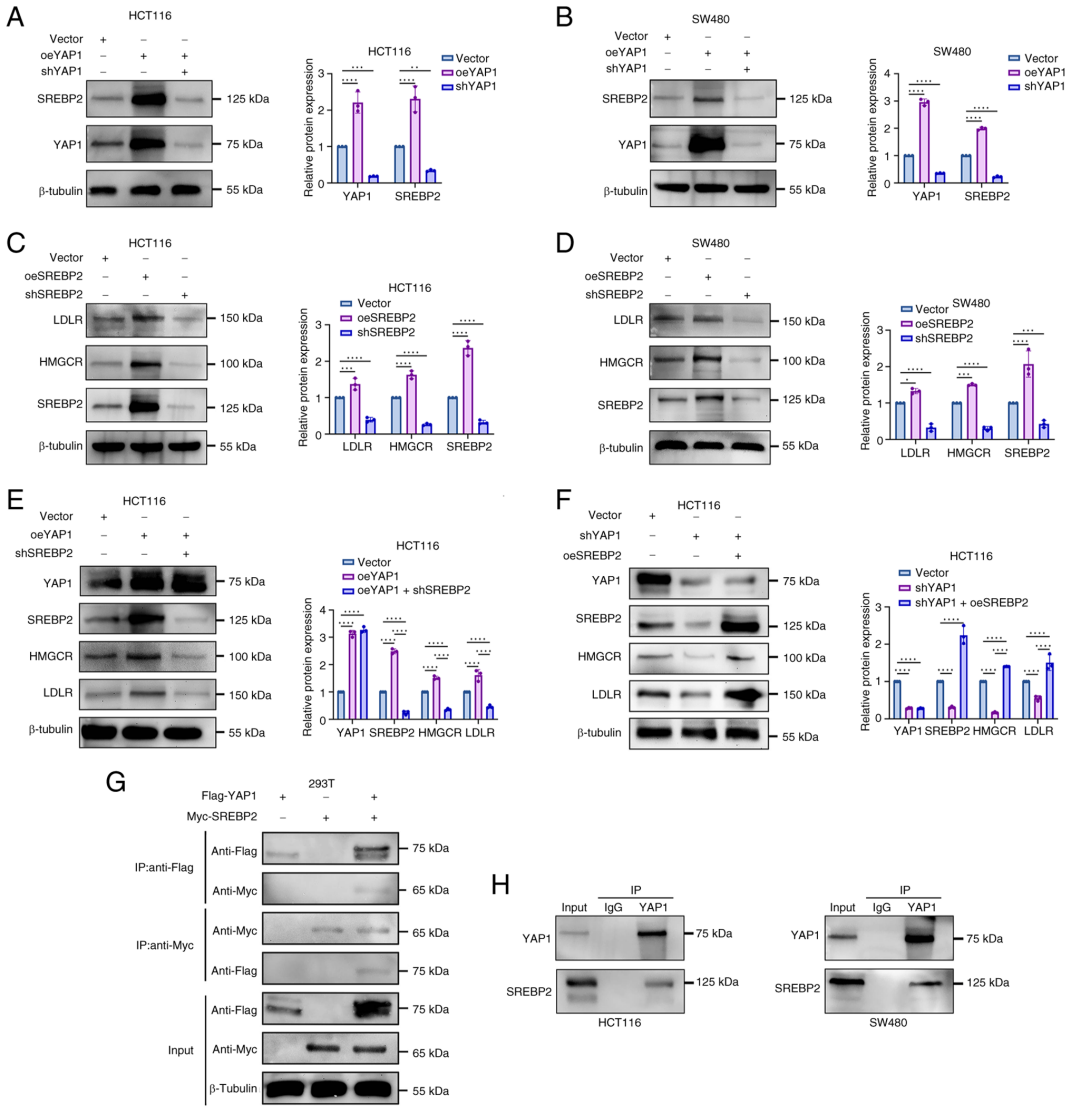
YAP1 co-operates with SREBP2 to modulate transcription of cholesterol metabolism genes. Western blot analysis of SREBP2 levels following YAP1 knockdown in (A) HCT116 and (B) SW480 cells. Assessment of downstream target gene expression upon SREBP2 modulation in (C) HCT116 and (D) SW480 cells. (E) Rescue assay demonstrated the increase in cholesterol metabolism-related molecules in YAP1-overexpressing HCT116 cells was inhibited upon SREBP2 knockdown. (F) Restoration of cholesterol metabolism-related protein levels by SREBP2 overexpression in YAP1-knockdown HCT116 cells. β-Tubulin was used as the loading control. (G) Co-immunoprecipitation of 293T cells co-expressing YAP1-Flag and SREBP2-Myc, using anti-Flag or anti-Myc antibodies. (H) Endogenous YAP1-SREBP2 interaction in HCT116 and SW480 cells was assessed by immunoprecipitation with an anti-YAP1 antibody followed by western blotting. Normal IgG was used as the negative control. All data are represented as mean ± standard deviation of at least three repeats. ^*^P<0.05, ^**^P<0.01, ^***^P<0.001, ^****^P<0.0001. YAP1, yes-associated protein 1; SREBP2, sterol regulatory element-binding protein 2; IgG, immunoglobulin G.

SREBP2, acting as a principal transcriptional regulator of cholesterol metabolism, is recognized for maintaining cholesterol homeostasis. This is achieved by modulating genes involved in the sterol biosynthetic, such as *HMGCR*, and genes critical for cholesterol uptake, such as *LDLR*. In the present study, the potential influence of YAP1 on SREBP2 was explored. Using western blotting, it was found that YAP1 overexpression markedly increased SREBP2 protein levels, whereas YAP1 knockdown resulted in a partial reduction of SREBP2 protein expression in HCT116 and SW480 cells (Fig. 4A and B). SREBP2 knockdown or overexpression did not affect YAP1 expression (Fig. S4A and B), confirming that SREBP2 was a downstream effector of YAP1. Conversely, SREBP2-knockdown CRC cells exhibited a significant decrease in HMGCR and LDLR expression (Fig. 4C and D). To investigate whether YAP1's regulation of HMGCR and LDLR was mediated through SREBP2, a rescue experiment was performed. Knockdown of SREBP2 abrogated the effect of YAP1 overexpression on increasing both HMGCR and LDLR protein expression levels (Fig. 4E).

SREBP2 was next overexpressed in the YAP1-knockdown CRC cells. Overexpression of SREBP2 restored the protein expression level of HMGCR and LDLR, which had been previously downregulated by YAP1 knockdown (Fig. 4F). Next, whether YAP1 reciprocally interacted with SREBP2 was explored. In 293T cells, YAP1-Flag and SREBP2-Myc plasmids were co-expressed, followed by immunoprecipitation and immunoblotting. The results showed that YAP1 was co-precipitated with SREBP2, suggesting a protein-protein interaction between them (Fig. 4G). In addition, SREBP2 was also detected in the YAP1 immunoprecipitate, the lysates derived from both HCT116 and SW480 cells (Fig. 4H). Collectively, these findings revealed that YAP1 physically interacted with SREBP2 and functioned as an upstream regulator that enhanced SREBP2 expression and transcriptional activity, leading to increased expression of its downstream targets, HMGCR and LDLR, in the cholesterol biosynthesis pathway.

### YAP1 promotes the nuclear translocation of SREBP2

**Figure 5:**
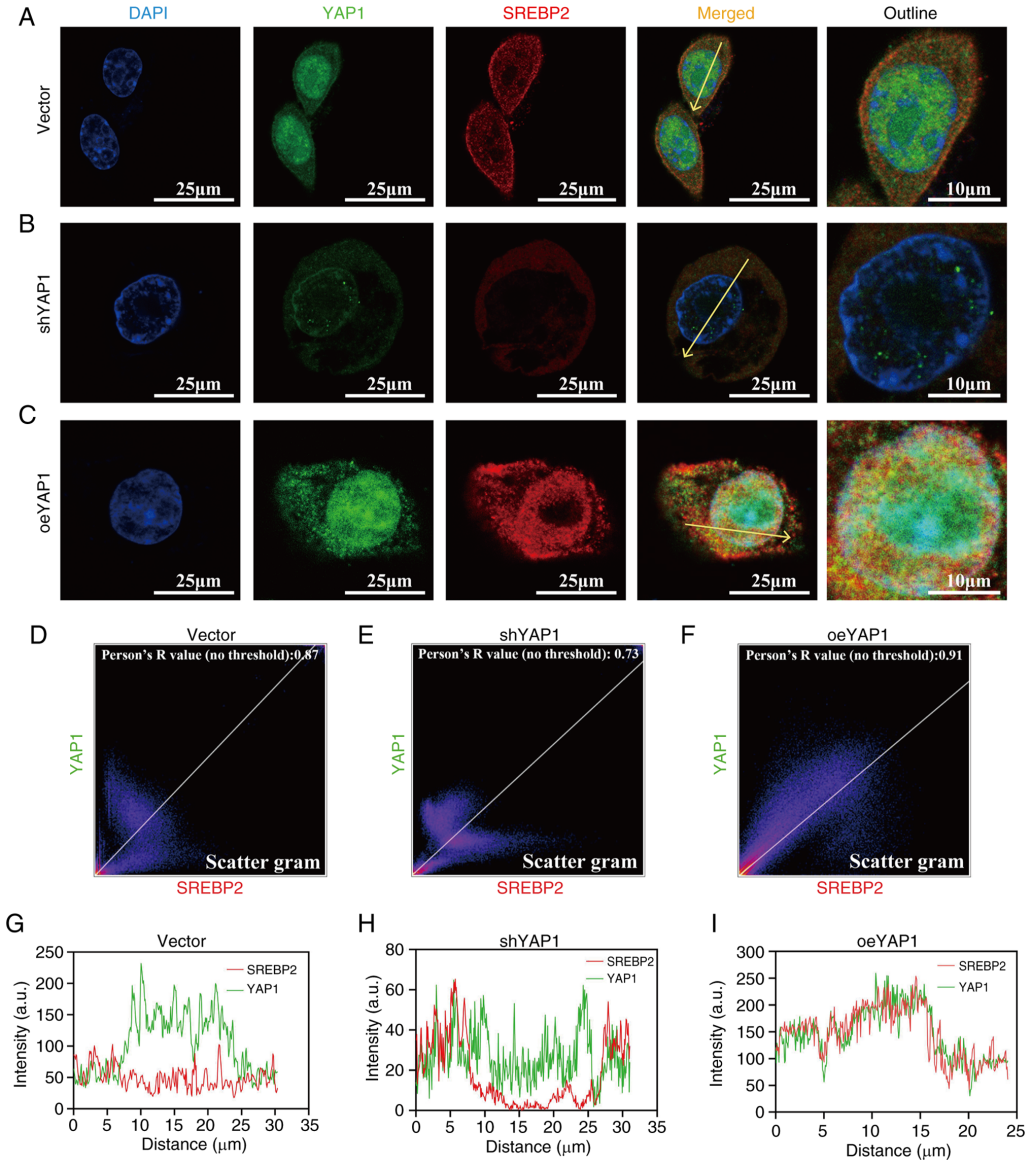
YAP1 promotes SREBP2 nuclear translocation in CRC cells. Representative confocal images showing YAP1 and SREBP2 co-localization in the cytoplasm and nucleus of HCT116 cells under (A) control conditions, (B) following YAP1 knockdown and (C) following YAP1 overexpression. Nuclei were counterstained with DAPI. The yellow area in the merged images indicated co-localization of YAP1 and SREBP2. Scale bars, 25 *μ*m (overview) and 10 *μ*m (inset). Pearson's correlation coefficient analysis quantifying the degree of co-localization between YAP1 and SREBP2 in (D) empty vector, (E) shYAP1 and (F) oeYAP1 cells. Fluorescence intensity profiles along the indicated yellow arrows represent the spatial distribution of YAP1 and SREBP2 signals in (G) control vector, (H) shYAP1 and (I) oeYAP1 cells. YAP1, yes-associated protein 1; SREBP2, sterol regulatory element-binding protein 2; CRC, colorectal cancer; shYAP1, short hairpin RNA targeting YAP1; oeYAP1, YAP1 overexpression.

Given that the functions of SREBP2 were dependent on its subcellular localization, whether YAP1 regulated its translocation from the cytoplasm to the nucleus was assessed. To directly visualize this distribution in cells, an IF experiment was conducted. In control cells, YAP1 was predominantly localized in the nucleus, whereas SREBP2 was enriched in the cytoplasm, with partial co-localization at the nuclear periphery (Fig. 5A). Upon YAP1 knockdown, not only did the nuclear signal of YAP1 decrease, but the fluorescence intensity of SREBP2 also decreased, accompanied by a marked reduction in its nuclear accumulation (Fig. 5B). Conversely, overexpression of YAP1 remarkably enhanced the fluorescence intensity of both proteins and markedly promoted the nuclear enrichment of SREBP2 (Fig. 5C). Quantitative co-localization analysis substantiated these observations. Pearson's correlation coefficient analysis revealed that the degree of co-localization between YAP1 and SREBP2 was diminished upon YAP1 knockdown compared to control conditions, whereas YAP1 overexpression enhanced their co-localization (Fig. 5D-F). The concordance between the *in situ* co-localization and the co-immunoprecipitation data (Fig. 4E and F) validated the interaction between these two proteins.

Fluorescence intensity analysis was performed along the indicated arrows in Fig. 5A-C to assess the spatial distribution of YAP1 and SREBP2 signals. Consistently, only partial cytoplasmic co-localization of YAP1 and SREBP2 signals was observed in both control and YAP1-silenced HCT116 cells (Fig. 5G and H). By contrast, YAP1 overexpression induced a pronounced redistribution, characterized by tightly overlapping nuclear intensity curves for both proteins and a significant increase in nuclear SREBP2 accumulation (Fig. 5I). These findings indicate that YAP1 not only upregulates SREBP2 expression but also physically interacts with it, potentially influencing its nuclear translocation.

**Figure 6:**
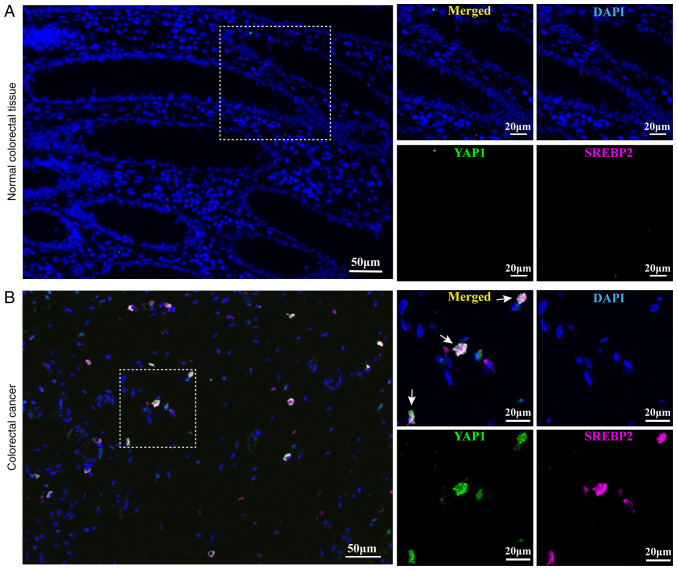
YAP1 and SREBP2 exhibit nuclear co-localization in human CRC tissues. Representative immunofluorescence staining of YAP1 and SREBP2 in (A) normal colorectal mucosa and (B) CRC tissue. Nuclei were counterstained with DAPI. Higher magnification views of the boxed areas are shown in the insets. White arrows indicate regions of YAP1-SREBP2 co-localization, appearing as yellow signals in the merged images. Scale bars, 50 *μ*m (overview) and 20 *μ*m (inset). Immunofluorescence staining was performed on tissue samples from three independent patients, yielding similar results. YAP1, yes-associated protein 1; SREBP2, sterol regulatory element-binding protein 2; CRC, colorectal cancer.

To validate the clinical relevance of this interaction, IF double-staining for YAP1 and SREBP2 was performed in three pairs of human CRC tissues and adjacent normal tissues. The normal colorectal tissues exhibited minimal fluorescence signal for both YAP1 and SREBP2, with no discernible co-localization (Fig. 6A). By contrast, CRC tissues showed markedly elevated expression of both proteins (Fig. 6B). Importantly, strong nuclear co-localization of YAP1 and SREBP2 was observed in cancer cells, as evidenced by the yellow signals in the merged images (Fig. 6B). These observations corroborated the *in vitro* findings that YAP1 facilitated SREBP2 nuclear translocation and provided critical translational evidence supporting the presence of a YAP1-SREBP2 axis in human CRC.

### Active YAP1 directly drives SREBP2 nuclear translocation independent of transcriptional upregulation

**Figure 7:**
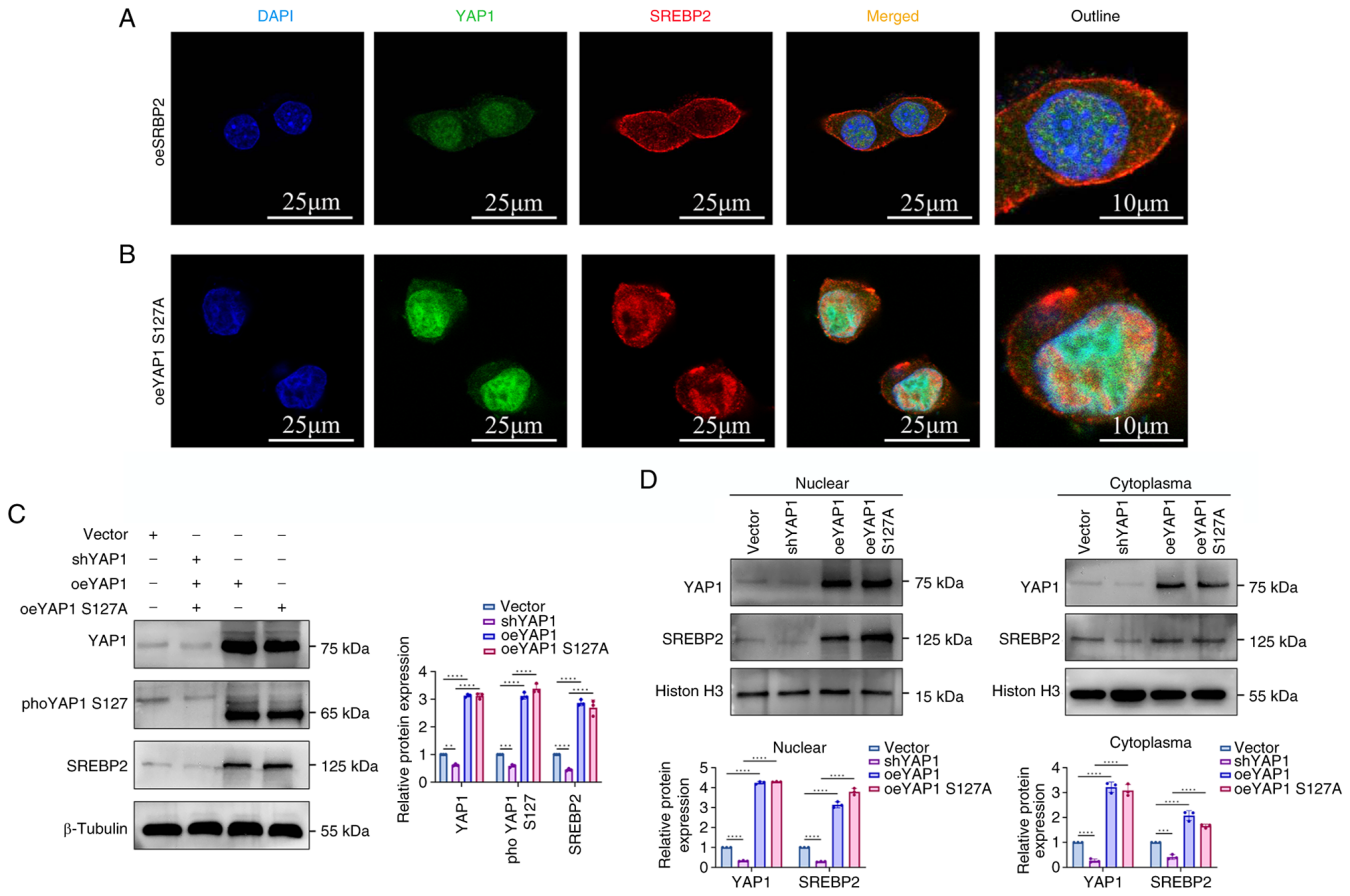
YAP1 S127A drives SREBP2 nuclear translocation independently of transcriptional upregulation. Representative immunofluorescent images depicting the distribution of YAP1 S127A and SREBP2 in HCT116 cells (A) overexpressing SREBP2 or (B) YAP1 S127A. Nuclei were stained with DAPI. Scale bars, 25 *μ*m (overview) and 10 *μ*m (inset). (C) Western blotting confirmed that YAP1 S127A overexpression enhanced SREBP2 expression and promoted its nuclear translocation *in vitro*. (D) Subcellular fractionation assay assessing SREBP2 distribution in cytoplasmic and nuclear compartments. Fraction purity was verified by western blotting for β-Tubulin (cytoplasmic control) and Histone H3 (nuclear control). All data are presented as the mean ± standard deviation of at least three repeats. ^**^P<0.01, ^***^P<0.001, ^****^P<0.0001. YAP1, yes-associated protein 1; SREBP2, sterol regulatory element-binding protein 2.

To further elucidate the mechanism by which YAP1 promoted SREBP2 nuclear translocation, whether YAP1 increased nuclear SREBP2 levels primarily through indirect upregulation of its total protein expression, or whether YAP1 played a more direct role in facilitating the translocation process itself, beyond its effect on SREBP2 expression, was assessed. S127 phosphorylation triggers YAP1 retention in the cytoplasm and subsequent proteasomal degradation, blocking its nuclear entry (7). Due to this regulatory mechanism, the S127A mutant is commonly employed as a constitutively active variant of YAP1. HCT116 cells were transfected with plasmids overexpressing YAP1 S127A or SREBP2. In cells overexpressing SREBP2, the protein was predominantly localized at the cell membrane and failed to accumulate efficiently in the nucleus, indicating that increasing SREBP2 protein levels alone was insufficient to effectively drive its nuclear translocation (Fig. 7A). By contrast, the overexpression of YAP1 S127A markedly enhanced the nuclear translocation of SREBP2, demonstrating that the transcriptional co-activator function of YAP1 was required for efficient SREBP2 nuclear translocation (Fig. 7B). In agreement with the results of IF experiments, western blotting showed that YAP1 overexpression, especially YAP1 S127A overexpression, induced rapid accumulation of the nuclear isoform of SREBP2 in the nucleus, while YAP1 knockdown suppressed the nuclear accumulation of SREBP2 (Fig. 7C and D). Taken together, these results reinforced the hypothesis that YAP1 activation served as a pivotal factor modulating the nuclear accumulation of SREBP2, independent of its role in total protein upregulation.

### YAP1 regulates CRC growth through a SREBP2-dependent cholesterol metabolism

**Figure 8:**
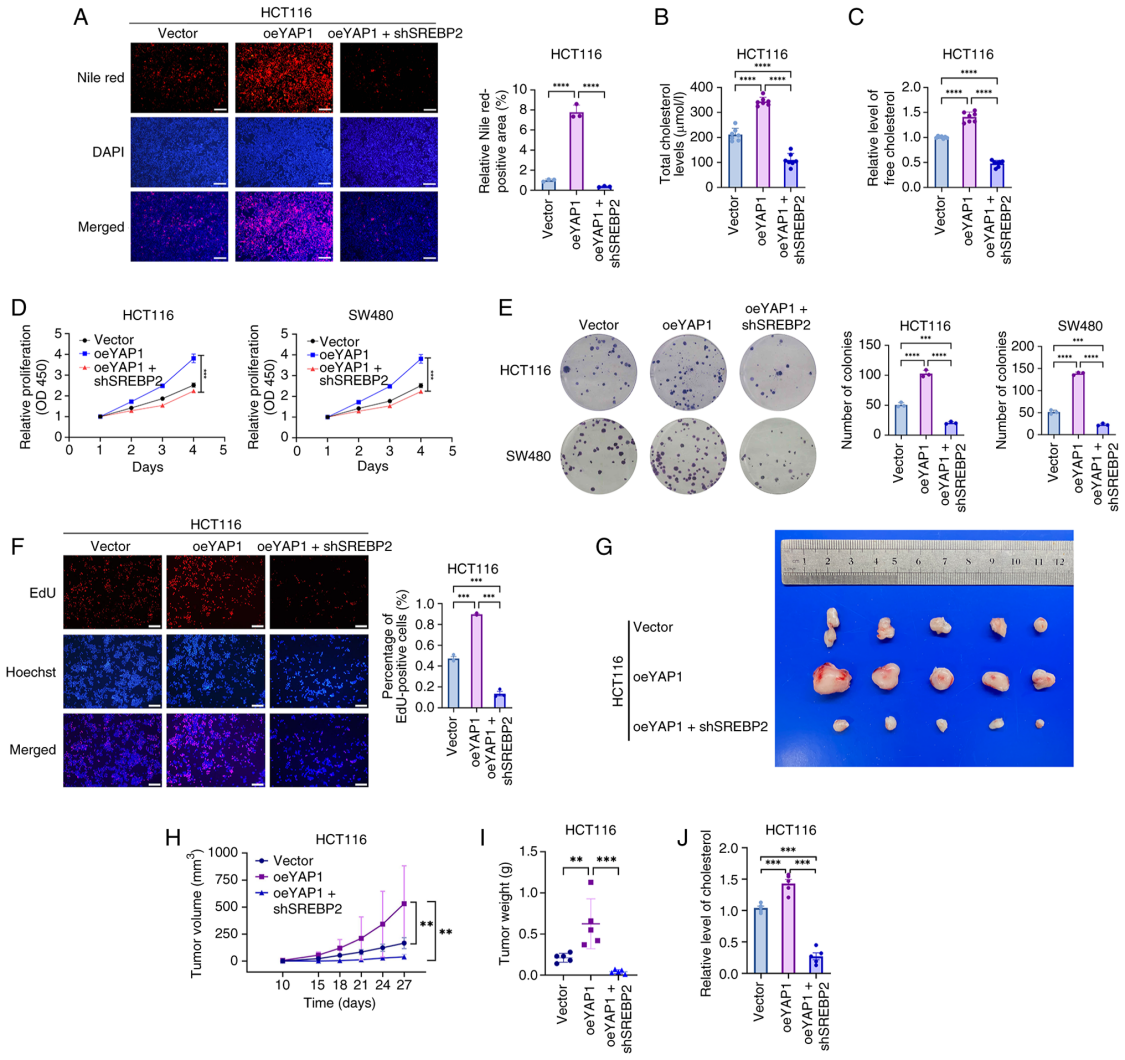
YAP1 promotes colorectal carcinogenesis through SREBP2-dependent cholesterol metabolism. (A) Lipid droplet visualization by Nile Red staining in YAP1-overexpressing HCT116 cells with or without SREBP2 knockdown. The right panel shows quantification of the relative fluorescence area. Scale bar, 100 *μ*m. Quantification of (B) total and (C) free cholesterol in YAP1-overexpressing HCT116 cells following SREBP2 knockdown. (D) The viability of YAP1-overexpressing HCT116 cells or SW480 cells was reduced following SREBP2 knockdown. (E) Colony formation in YAP1-overexpressing HCT116 cells or SW480 cells was reduced following SREBP2 knockdown. Representative images and quantitative analysis of the number of colonies formed are shown. (F) EdU incorporation assays revealed reduced proliferation following SREBP2 knockdown in YAP1-overexpressing HCT116 and SW480 cells. The right panel shows quantification of EdU-positive cells. Scale bar, 250 *μ*m. *In vivo* tumor growth following subcutaneous injection of empty vector, oeYAP1, or oeYAP1+shSREBP2 HCT116 cells into nude mice (n=5 per group). (G) Gross appearance of tumors. (H) Tumor volume growth curves over 27 days. (I) Tumor weight on day 27 post-transplantation. (J) Cholesterol concentration in tumor tissues. ^**^P<0.01, ^***^P<0.001, ^****^P<0.0001. YAP1, yes-associated protein 1; SREBP2, sterol regulatory element-binding protein 2; oeYAP1, YAP1 overexpression; shSREBP2, short hairpin RNA targeting SREBP2.

To functionally validate the YAP1-SREBP2 axis in CRC, whether SREBP2 was required for YAP1-driven cholesterol accumulation and tumorigenesis was investigated. SREBP2 expression was knocked down in YAP1-overexpressing HCT116 cells using an shRNA vector targeting SREBP2. Subsequently, cholesterol content and lipid droplet formation were evaluated. The overexpression of YAP1 markedly enhanced both lipid droplet accumulation and cellular cholesterol levels, whereas concomitant SREBP2 knockdown abolished these effects (Fig. 8A-C).

Considering the established link between cholesterol metabolism and tumor progression, whether the YAP1-SREBP2 axis contributed to CRC cell growth *in vitro* was further examined. To address this point, cell viability and colony formation capacity were assessed. SREBP2 knockdown reversed the increase in cell proliferation and colony formation induced by YAP1 overexpression (Fig. 8D and E). In parallel, EdU incorporation assays further confirmed that SREBP2 knockdown eliminated the increase in proliferation conferred by YAP1 overexpression (Fig. 8F). These findings indicated that the SREBP2-dependent cholesterol metabolic pathway was essential for YAP1 to regulate CRC cell growth.

Next, observations were validated *in vivo*. Xenograft tumors derived from YAP1-overexpressing HCT116 cells exhibited a markedly larger volume and weight compared to controls. Notably, this tumor-promoting effect was abrogated when SREBP2 was knocked down in YAP1-overexpressing cells (Fig. 8G-I). Correspondingly, SREBP2 knockdown also reversed the effects of YAP1-overexpression on enhancing cholesterol concentration in CRC tissues (Fig. 8J).

Together, these results showed that YAP1 promoted colorectal tumorigenesis and progression by modulating SREBP2 nuclear translocation and expression, thereby enhancing cholesterol metabolism.

## Discussion

**Figure 9:**
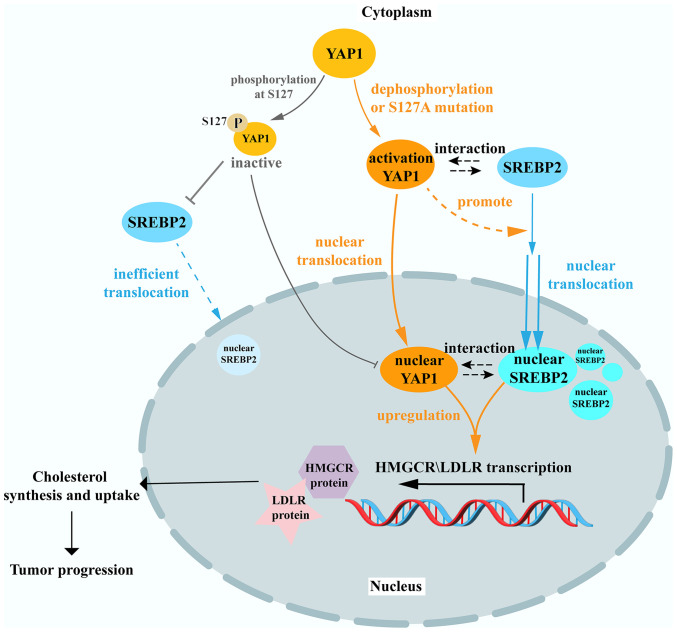
The predicted molecular mechanism by which YAP1-mediated regulation of cholesterol metabolism in CRC. Under basal conditions, phosphorylated YAP1 (Ser127) is retained in the cytoplasm in an inactive state, resulting in inefficient SREBP2 nuclear translocation. Upon activation (dephosphorylation or S127A mutation), YAP1 translocates to the nucleus, where it interacts with and facilitates the efficient nuclear translocation of SREBP2. Nuclear YAP1 and SREBP2 synergistically upregulate cholesterol metabolism genes HMGCR and LDLR, enhancing cholesterol synthesis and uptake to drive CRC progression. YAP1, yes-associated protein 1; CRC, colorectal cancer; SREBP2, sterol regulatory element binding protein 2; HMGCR, 3-hydroxy-3-methylglutaryl-CoA reductase; LDLR, low-density lipoprotein receptor; P, phosphorylation.

Although clinical management of CRC has improved, therapeutic outcomes for patients with CRC remain suboptimal. These limitations underscore the importance of elucidating the molecular mechanisms driving tumor initiation and disease progression (27). Cholesterol and its derivatives, essential structural components of various cell membranes, are key factors that influence several cellular processes and promote cancer development. Emerging evidence suggests that targeting dysregulated cholesterol metabolism is an attractive therapeutic target in oncology (28). Although the association between YAP1 and cellular metabolism is well established, the molecular mechanisms that regulate cholesterol metabolism in CRC remain poorly understood. The present study showed that YAP1 increased SREBP2 transcriptional activity and physically interacted with it to promote its nuclear translocation. This dual regulation resulted in the upregulation of cholesterol metabolic genes, including HMGCR and LDLR, ultimately facilitating tumorigenesis and proliferation in CRC (Fig. 9).

The canonical SREBP2 activation pathway relies on sterol deficiency-triggered, SCAP-mediated proteolytic processing, ultimately generating mature SREBP2 (N-SREBP2) (29). As a transcription factor, N-SREBP2 drives the transcriptional program of cholesterol metabolism-related genes by recruiting epigenetic regulators and transcriptional co-activator complexes (22). In the present study, multi-omics analysis revealed that silencing YAP1 in CRC cells specifically downregulated genes responsible for cholesterol biosynthesis and uptake, leading to reduced cellular cholesterol levels. These results revealed that YAP1 functions as a key modulator in maintaining cholesterol metabolic homeostasis in CRC. However, the relative contributions of cholesterol biosynthesis compared with exogenous uptake were not distinguished in the present study. To directly verify YAP1-mediated transcriptional changes, isotope-tracing techniques could be employed to measure alterations in cholesterol flux in YAP1-inhibited CRC cells in future studies. Notably, the changes in Nile Red staining observed in this study reflect alterations in both cholesteryl ester and triglycerides, implying a broader role for YAP1 in neutral lipid metabolism beyond cholesterol alone. Nevertheless, the present data confirmed that cholesterol metabolism is the primary and functionally relevant outcome of YAP1-mediated SREBP2 activation in promoting CRC malignant progression.

Mechanistically, co-immunoprecipitation, fluorescence co-localization and rescue experiments established that YAP1 specifically interacted with SREBP2 in CRC cells, predominantly co-localizing within the nucleus. Appropriate cytoplasmic-nuclear translocation of proteins is crucial for cellular homeostasis and its dysregulation is commonly observed in carcinoma (30,31). The results of the present study further revealed that the nucleocytoplasmic distribution of SREBP2 was closely associated with YAP1 expression levels: Silencing YAP1 inhibited nuclear translocation of SREBP2 isoforms, while overexpressing YAP1, particularly the constitutively active mutant YAP1 S127A, markedly promoted SREBP2 nuclear accumulation. By contrast, SREBP2 overexpression alone was insufficient to alter its subcellular localization, indicating that elevated SREBP2 protein levels were not the driving force behind its nuclear translocation. These findings highlighted YAP1 as a critical upstream regulator that directly facilitates SREBP2 nuclear translocation, rather than exerting this effect indirectly through transcriptional upregulation of SREBP2. A notable observation from the rescue experiments was that SREBP2 knockdown in YAP1-overexpressing cells suppressed cholesterol metabolism and cell growth to levels below those of control cells, further underscoring a dual role of SREBP2, which functions both as a downstream effector of YAP1-driven oncogenic signaling and as an independent regulator of basal cholesterol homeostasis. Together, these findings revealed an alternative, oncogene-driven activation mechanism for SREBP2 in CRC that operated independently of the classical sterol-sensing pathway. However, it remains unclear whether YAP1 interacts with SREBP2 directly or indirectly through cofactors. The present study did not identify the specific interaction domains or phosphorylation events mediating their association. These aspects represent limitations of the current work, and future studies are warranted to clarify this regulatory mechanism.

The SREBP transcription factor family consists of three members: SREBP-1a, SREBP-1c and SREBP-2, each playing distinct role in regulating lipid metabolism. SREBP-1a is involved in lipid biosynthesis and growth, while SREBP-1c regulates fatty acid synthesis and energy homeostasis. SREBP-2, by contrast, primarily controls cholesterol metabolism (32,33). Accumulating evidence points to a functional link between YAP1 and the SREBP1-dependent lipogenic programs. In mouse hepatocytes, YAP1 may interact with SREBP1/SREBP2 to modulate lipogenesis and cholesterol synthesis, contributing to hyperlipemia and fatty liver development (23). In normal breast epithelial cells, YAP1 targets SGK1 to activate mTORC1, which, in turn, stimulates the SREBP1-regulated transcriptional program of lipogenesis (24). Furthermore, in CRC, FUT2 reprograms fatty acid metabolism by promoting YAP1 nuclear translocation and stabilizing mature SREBP-1, thereby facilitating metastasis (34). In contrast to the rapidly expanding knowledge of YAP1 in lipogenesis reprogramming, the relationship between YAP1 and SREBP2-driven cholesterol metabolism remains controversial. In hepatocellular carcinoma, silencing LATS inhibited SREBP2 activation and its nuclear translocation, suggesting a potential link between Hippo signaling and SREBP2 (31). Additionally, Pan *et al* (35) demonstrated that YAP-mediated ZMYND8 promoted mediator complex recruitment by interacting with SREBP2, upregulating cholesterol biosynthesis gene expression, and subsequently driving intestinal stem cell tumorigenesis. Wang *et al* (36) recently identified a STAT1-YAP1 feedforward circuit that transcriptionally activated SREBF2 via TEAD4 in KRAS-mutant CRC cells, further linking YAP1 to the mevalonate pathway. However, while Pan *et al* (35) utilized CRC organoids and focused on stem cell differentiation, and Wang *et al* (36) emphasized transcriptional regulation of SREBF2, the present study revealed a distinct post-transcriptional mechanism in two human CRC cell lines, HCT116 and SW480. It was demonstrated that YAP1 physically interacted with SREBP2 protein and promoted its nuclear translocation, independent of SREBF2 mRNA upregulation. Thus, the central role of this protein-protein interaction in driving CRC cell proliferation was directly demonstrated, providing novel experimental evidence and mechanistic insights in this field.

The findings of the present study identify a previously unrecognized metabolic function of YAP1 in CRC, implicating the YAP1-SREBP2-HMGCR/LDLR axis as a potential therapeutic target. Previously, YAP1 inhibitors such as verteporfin have demonstrated promising antitumor efficacy in preclinical CRC models (37,38). Studies have indicated that verteporfin exerts its effects through multiple mechanisms, including remodeling cancer-associated fibroblasts, inhibiting cell proliferation and invasion and reversing chemotherapy resistance (39,40). YAP1 has also been implicated in immune evasion, as it upregulates PD-L1 and drives M2 macrophage polarization, thereby contributing to an immunosuppressive microenvironment that dampens immunotherapy responses (41). Similarly, cholesterol homeostasis is closely related to T-cell function and antitumor immunity (42). Therefore, targeting the YAP1-SREBP2 axis may reverse the immunosuppression by reducing tumor cholesterol levels. These observations support the rationale for combining YAP1-targeted agents with immune checkpoint inhibitors, including programmed cell death protein 1 (PD-1)/programmed death ligand 1 (PD-L1) inhibitors. However, the present study was limited by its use of immunodeficient nude mice, which preclude assessment of how the YAP1-SREBP2 axis modulates adaptive immune response. The role of this pathway in immune regulation should be further explored in immunocompetent systems, such as immunocompetent syngeneic mouse models. This would enable assessment of its impact on immune surveillance, tumor-immune crosstalk, and responses to combination immunotherapies.

Furthermore, the identification of the YAP1-SREBP2 signaling pathway offers a novel rationale for combined metabolic intervention. Notably, emerging evidence suggests a bidirectional feedback loop between the mevalonate pathway and YAP/TAZ activity. Statins, as classic HMGCR inhibitors, have demonstrated potential for the chemoprevention of CRC (43). Recent advances have shown that statins not only reduce cholesterol synthesis but also suppress YAP/TAZ activation by disrupting protein geranylgeranylation (44). Conversely, YAP1 activation promotes the expression of mevalonate pathway genes, creating a positive feedback circuit that may sustain oncogenic signaling (35). However, long-term statin use may trigger compensatory feedback activation of SREBP2, thereby limiting therapeutic efficacy (45,46). This reciprocal regulation provides a strong rationale for combining YAP1 inhibitors with statins as a dual-blockade strategy against CRC: While the statin inhibits cholesterol synthesis, the YAP1 inhibitor transcriptionally suppresses the SREBP2-driven compensatory metabolic reprogramming, which may yield synergistic anti-tumor effects. Although the present study demonstrated the functional significance of the YAP1-SREBP2 axis, its clinical translation requires additional validation. Preclinical pharmacological studies using targeted agents in CRC models are needed to confirm the potential therapeutic value. Concurrently, prospective validation in well-annotated independent cohorts with standardized treatment records, comorbidities, and long-term follow-up represents an essential next step to confirm the clinical utility of YAP1 and facilitate its translation into precision oncology strategies.

In conclusion, the present study demonstrated that YAP1 promoted CRC progression by reprogramming cholesterol metabolism through SREBP2 activation. This oncogene-directed metabolic pathway not only deepens our understanding of the biological functions of Hippo signaling, but also establishes a crucial molecular foundation for developing metabolism-targeted therapies, especially combination strategies, against CRC.

## Supplementary Data



## Figures and Tables

**Table I:** Primer pairs used for quantitative PCR.

Genes	Forward primer sequence (5'-3')	Reverse primer sequence (5'-3')
*YAP1*	TCGTTTTGCCATGAACCAGA	GGCTGCTTCACTGGAGCACT
*SREBP2*	GGAGAAAGGCGGACAACC	CAGAGTCAATGGAGTAGGG
*HMGCR*	AGAAGAAAATAAGCCCAATC	TATCCAGCGACTGTGAGC
*HMGCS1*	TTCCCCAGGGTTCAATAG	GCCGAGCGTAAGTTCTTC
*LDLR*	CATGAGCGATGAAGTTGG	GGTGAAGAAGAGGTAGGC
*ABCA1*	CCCTGGGTGTCAGTAATA	TGACATTCAGGAAAGAGC
*ABCG*	AGGGACCTTTCCTATTCG	CATGACTGGAGGGTTGTT
*CYP7A1*	GTTGTCTATGGCTTATTCTTG	GTCGTTTATGTTTTCAGTGGTA
*MYLIP*	AGAGTCAAGTTCTTCGTGG	TTCTTTCCTGACTGGGTG
*ACTIN*	CACCATTGGCAATGAGCGGTTC	AGGTCTTTGCGGATGTCCACGT

**Table II:** Antibodies used for western blotting, co-immunoprecipitation, immunofluorescence and immunohistochemistry.

Antibody	Catalogue number	Supplier	Experiment	Dilution
Anti-YAP1	66900-1-Ig	Proteintech Group, Inc.	WB	1:10,000
			IHC	1:1,500
			IF	1:1,000
Anti-SREBF2	28212-1-AP	Proteintech Group, Inc.	WB	1:8,000
			IF	1:100
Anti-HMGCR	A19063	ABclonal Biotech Co., Ltd.	WB	1:1,000
Anti-LDLR	10785-1-AP	Proteintech Group, Inc.	WB	1:3,000
Anti-phospho-YAP1 S127	CABP0489	AssayGenie	WB	1:1,000
			IF	1:100
Anti-YAP1	13584-1-AP	Proteintech Group, Inc.	IF	1:200
Anti-SREBP2	sc-271615	Santa Cruz Biotechnology, Inc.	IF	1:300
Anti-Flag	AE005	ABclonal Biotech Co., Ltd.	WB	1:60,000
			IP	5 *μ*g/ml cell lysate
Anti-Myc	AE070	ABclonal Biotech Co., Ltd.	WB	1:80,000
			IP	5 *μ*g/ml cell lysate
FITC-conjugated antibody	GB22401/GB22403	Wuhan Servicebio Technology Co., Ltd.	IF	1:50
CY3-conjugated antibody	GB21301/GB21303	Wuhan Servicebio Technology Co., Ltd.	IF	1:100
Anti-β-Tubulin	66240-1-Ig	Proteintech Group, Inc.	WB	1:80,000
Anti-histone H3	68345-1-Ig	Proteintech Group, Inc.	WB	1:10,000
Anti-IgG	sc-515946	Santa Cruz Biotechnology, Inc.	IP	1:10,000

WB, western blotting; IHC, immunohistochemistry; IF immunofluorescence; YAP1, yes-associated protein 1; SREBF2, sterol regulatory element binding transcription factor 2; SREBP2, sterol regulatory element binding protein 2; HMGCR, 3-hydroxy-3-methylglutaryl-CoA reductase; LDLR, low-density lipoprotein receptor; IgG, immunoglobulin G; WB, western blotting; IHC, immunohistochemistry; IF, immunofluorescence; IP, immunoprecipitation.

**Table III:** Relationship between YAP1 staining intensity and clinicopathological characteristics in a CRC tissue microarray.

Features	YAP1 expression		P-value^a^
	High	Low	
Age (years; n=96)			
≤60	15	17	0.078
>60	42	22	
Sex (N=96)			
Male	30	22	0.715
Female	27	17	
Histological grade (n=94)			
Low grade	45	24	0.064
High grade	11	14	
Histological type (n=94)			
Conventional adenocarcinoma	49	27	0.047
Special subtype^b^	7	11	
T stage (n=93)			
T1-2	2	1	0.963
T3	31	19	
T4	24	16	
Pathological N stage (n=95)			
N0	32	23	0.346
N1a	5	9	
N1b	10	4	
N1c	1	0	
N2a	3	1	
N2b	5	2	
M stage (n=96)			
M0	54	39	0.269
M1	3	0	
Disease stage (n=94)			
I	2	1	0.526
II	30	21	
III	21	16	
IV	3	0	

Staging of the tumor was conducted in accordance with the AJCC 8th Edition recommendations.

a P-values were calculated using the Fisher's exact test, P<0.05 was considered statistically significant.

b Special histological subtype includes mucinous adenocarcinoma and signet-ring cell carcinoma. YAP1, yes-associated protein 1; CRC, colorectal cancer.

**Table IV:** Multivariate Cox regression analysis of factors associated with overall survival in a CRC tissue microarray.

Factor	HR	95%CI	P-value^a^
Sex (male vs. female)	0.630	0.331-1.200	0.160
Age, years (>60 vs. ≤60)	1.413	0.710-2.811	0.325
Disease stage (III, IV vs. I, II)	2.066	1.104-3.863	**0.023**
Histological grade (high grade vs. low grade)	1.160	0.557-2.416	0.692
Histological type (special subtype1^b^ vs. conventional adenocarcinoma)	1.755	0.755-4.076	0.191
YAP1 expression (high vs. low)	2.322	1.122-4.807	**0.023**

a P<0.05 was considered statistically significant. Bold values indicate statistically significant differences.

b Special histological subtype includes mucinous adenocarcinoma and signet-ring cell carcinoma. YAP1, yes-associated protein 1; CRC, colorectal cancer; HR hazard ratio; CI, confidence interval.

## Data Availability

The data generated in the present study may be found in the OMIX, China National Center for Bioinformation/Beijing Institute of Genomics, Chinese Academy of Sciences: https://ngdc.cncb.ac.cn/omix; under accession numbers OMIX015892 for RNA-seq data (https://ngdc.cncb.ac.cn/omix/preview/tEzn98RI); accession no. OMIX015935 for metabolomics data (https://ngdc.cncb.ac.cn/omix/preview/v6h8aZlZ). Raw FASTQ files are not available.
