# Supplementary material for: YAP1-mediated cytoplasmic-nuclear translocation of SREBP2 promotes colorectal cancer via regulation of cholesterol metabolism
Source: Int J Oncol. 2026 Jul 13;69(3):105. doi: 10.3892/ijo.2026.5918 (PMC13387130; doi:10.3892/ijo.2026.5918)

Figure S1. Overview of YAP1 immunohistochemistry on tissue microarrays. Black boxes in the layout indicate the CRC tissues. Image was obtained at x40 magnification. YAP1, yes-associated protein 1; CRC, colorectal cancer.

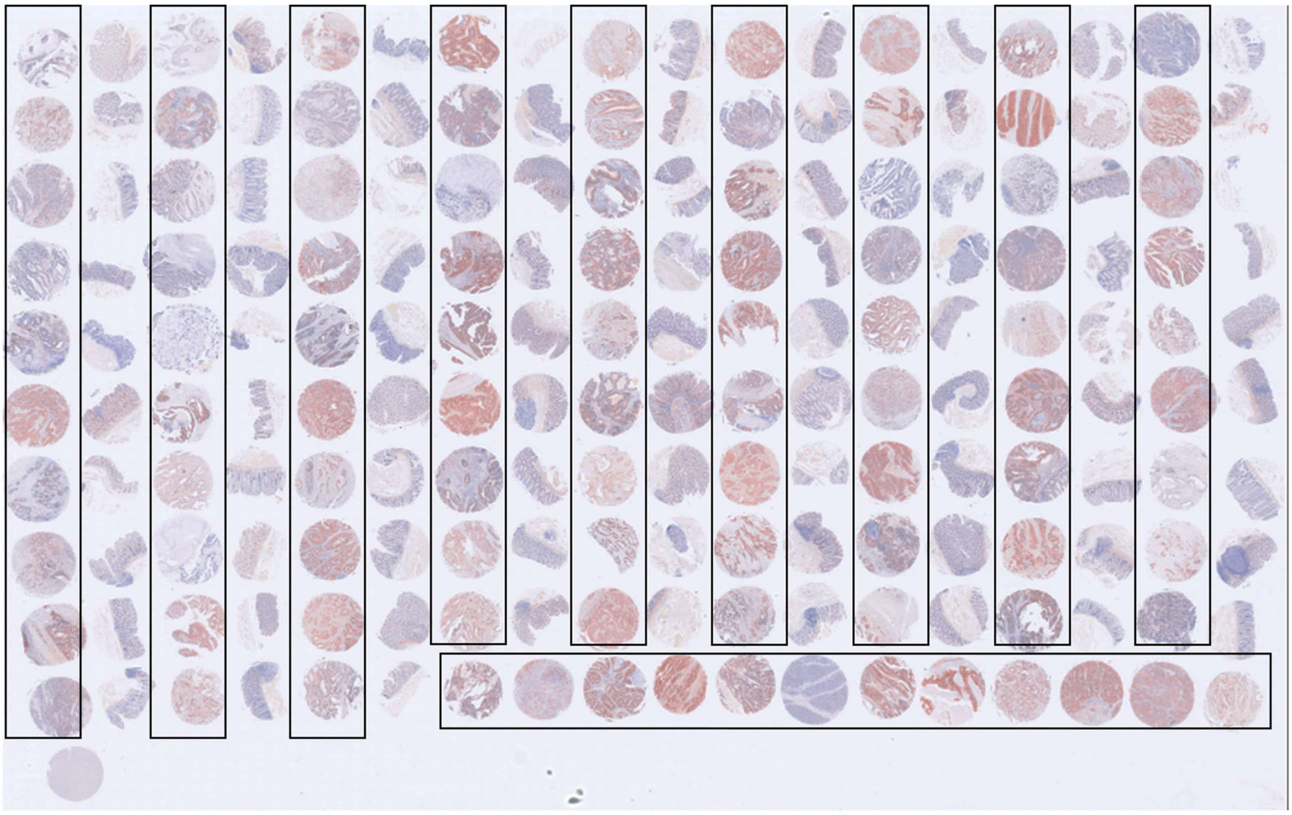

Figure S2. Multivariate Cox regression analysis of prognostic factors for overall survival in CRC tissue microarray. Forest plot showing adjusted HR for overall survival. The multivariate Cox proportional hazards regression model included YAP1 expression, sex, age, disease stage, histological grade, and histological type as covariates. Horizontal lines represent a 95% CI. HR>1 indicates increased risk of death. CRC, colorectal cancer; HR, hazard ratio; CI, confidence interval.

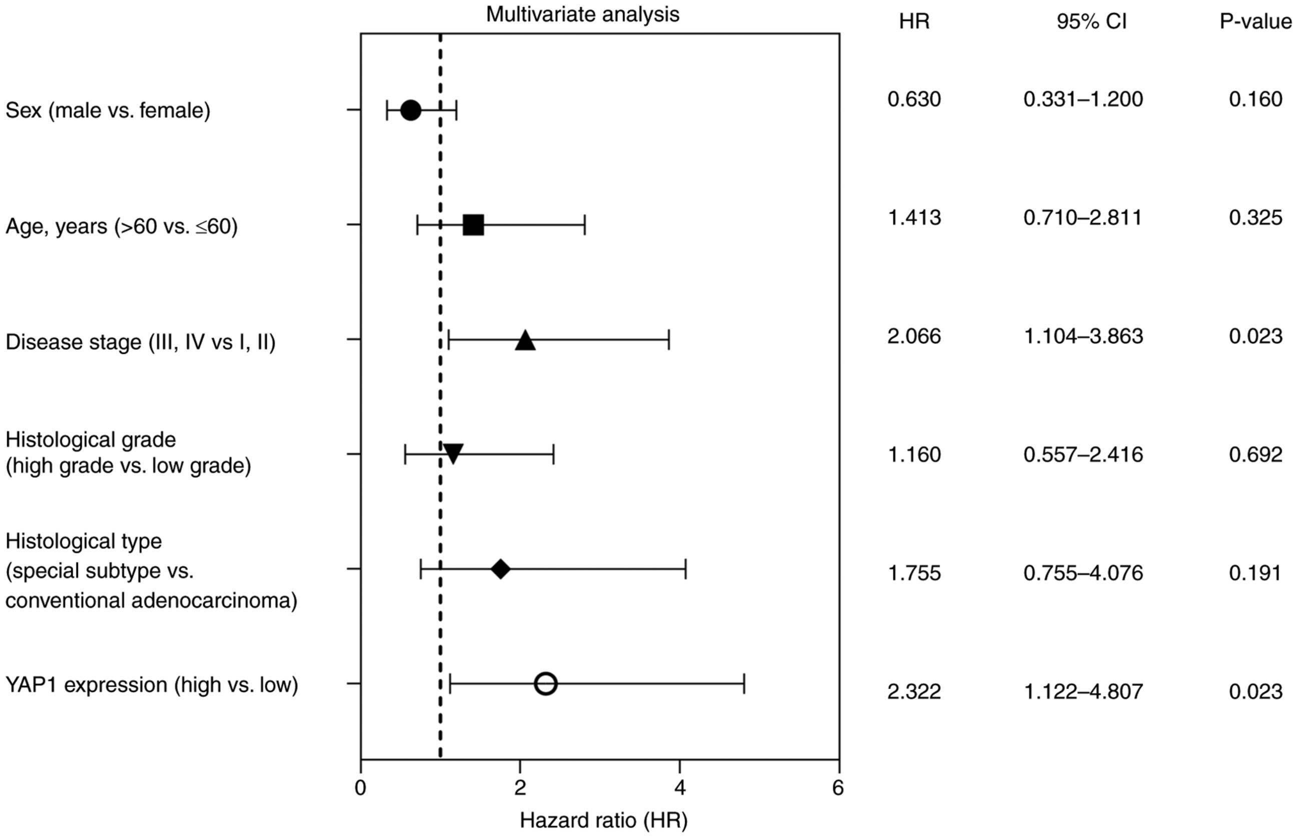

Figure S3. Stage-stratified survival analysis of YAP1 expression in a CRC tissue microarray. (A and B) Kaplan-Meier curves comparing overall survival between high- and low-YAP1 expression groups in patients with (A) early-stage (I-II) and (B) advanced-stage (III-IV) CRC. YAP1, yes-associated protein 1; CRC, colorectal cancer.

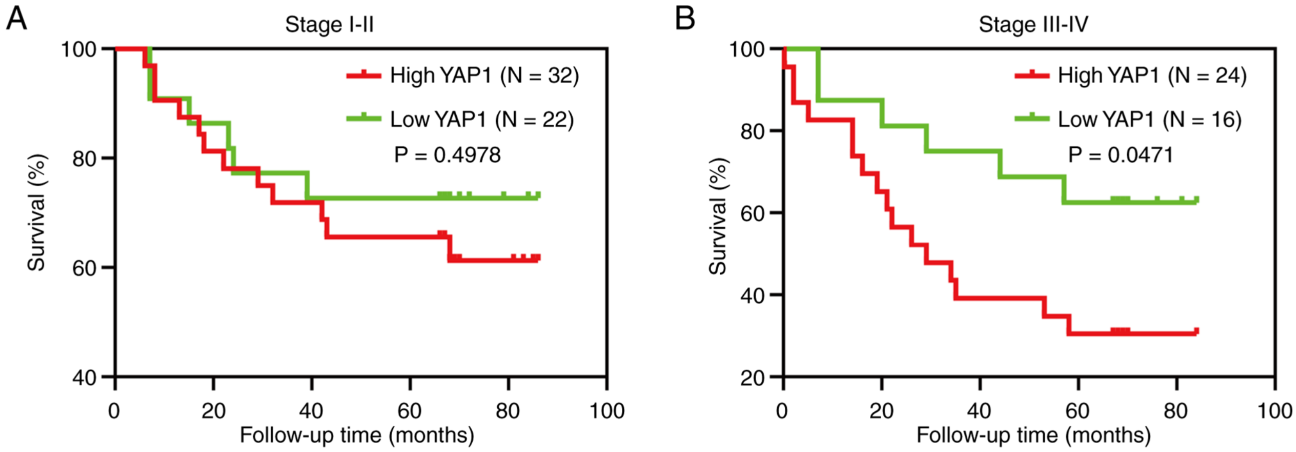

Figure S4. YAP1 expression is unaffected by SREBP2 modulation. Western blot analysis of YAP1 levels following SREBP2 knockdown in (A) HCT116 and (B) SW480 cells. β-Tubulin was used as the loading control. All data are represented as mean ± standard deviation of three repeats. **P<0.01, ***P<0.001, ****P<0.0001. ns, not significant; YAP1, yes-associated protein 1; SREBP2, sterol regulatory element-binding protein 2; oeSREBP2, SREBP2 overexpression; shSREBP2, short hairpin RNA targeting SREBP2.

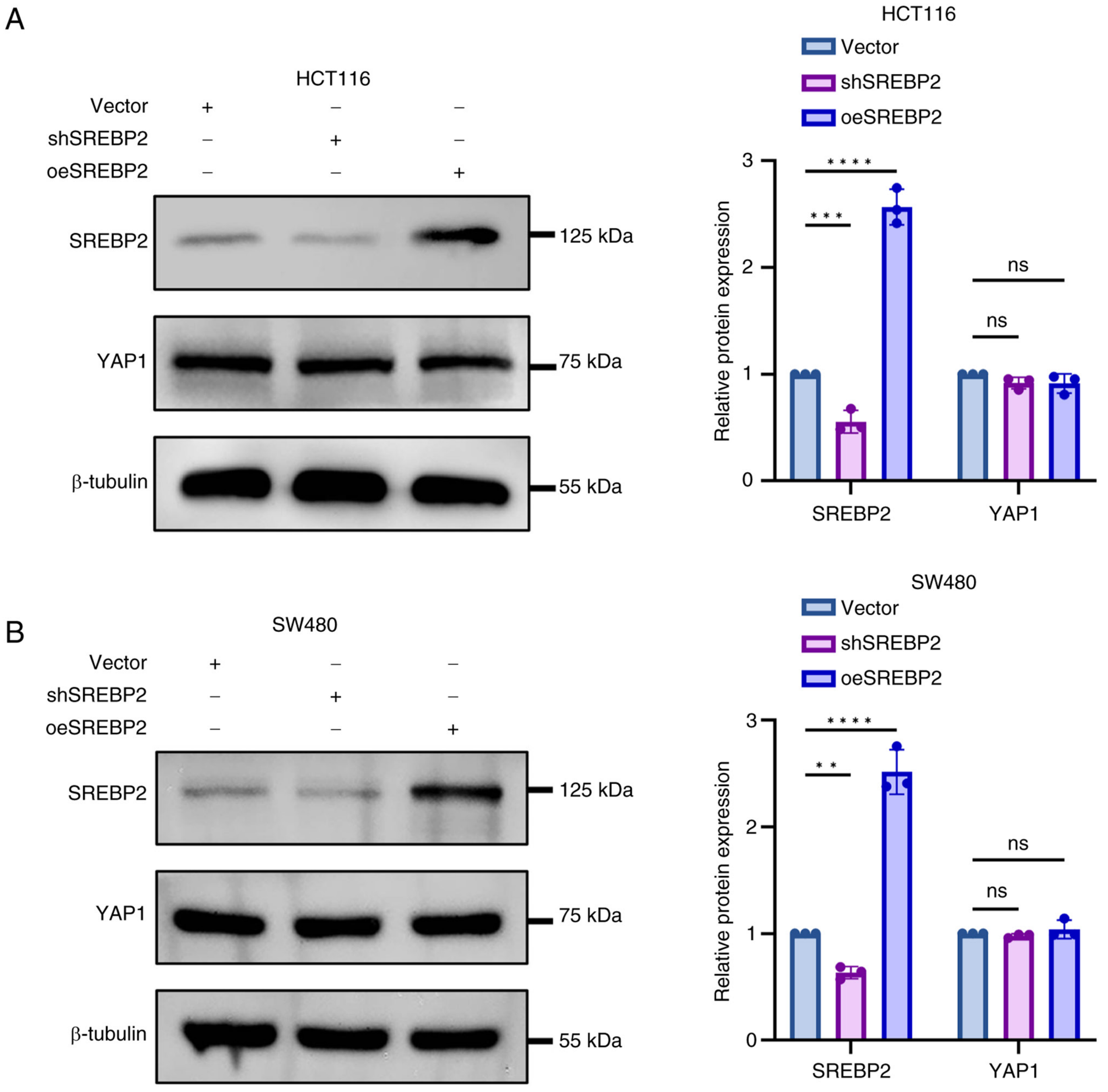

Supplement: Supplementary file 1 [file Supplementary_Data.pdf]
